# Supplementary material for: Lactobacillus Reuteri DSM 17938 (Limosilactobacillus reuteri) in Diarrhea and Constipation: Two Sides of the Same Coin?
Source: Medicina (Kaunas). 2021 Jun 23;57(7):643. doi: 10.3390/medicina57070643 (PMC8306447; doi:10.3390/medicina57070643)
Supplement: Supplementary file 1 [file medicina-57-00643-s001.zip › medicina-1237548-supplementary.pdf]

Table S1. Summary of studies on the role of *L. reuteri* in constipation and diarrhea.

Study	Patients	Probiotic (*L. reuteri* DSM 17938)	Duration	Results in *constipation*
Coccorullo et al [38]	44 infants (5–10 months old)<br>22 probiotics vs 22 placebo	1x 10^8 CFU/day drops of oily suspension	8 weeks	No therapeutic success in constipation<br>Higher frequency of bowel movements in probiotic group at 2-4-8 weeks. No difference in stool consistency
Ojetti et al [3]	20 adults (mean age 36.2 ± 13.7)	10^8 CFU in 1 capsule	4 weeks	Beneficial effect on chronic constipation, via a significant decrease of CH^4 production.
Riezzo et al [42]	56 patients	4×10^8 CFU (28 patients) vs 2×10^8 CFU (28 patients)	105 days	Useful in association therapy rather than as single-drug therapy in the management of constipation.
Indrio et al [39]	468 infants (first 3 months of life)<br>238 in probiotics groups vs 230 placebo	1 × 10^^{8} CFU/day drops of oily suspension	90 days	Improves constipation during the first 3 months of life.
Kubota et al [2]	60 (>6 months- <6 years old)<br>20 probiotics + placebo of MgO vs 19 probiotics + MgO vs 21 placebo of probiotics and MgO	10^^{8} CFU in 5 drops of a commercially available oil suspension, twice a day	4 weeks	*L. reuteri DSM 17938* was effective in functional constipation in young children. MgO caused an imbalance in the gastrointestinal microbiome, which was not reported in the group treated with this probiotic.
Ojetti et al [37]	40 adults (18M/22F, 35+/-15 years)<br>20 probiotics vs 20 placebo	10^8<br>CFU in tablets twice per day	4 weeks	Effective in improving bowel movement frequency in adult patients with functional constipation but no effect on stool consistency.
**Study**	**Patients**	**Probiotic (*L. reuteri*)**	**Duration**	**Results in *diarrhea***
Francavilla et al [18]	69 infants (6-36 months old)<br>35 probiotics vs 34 placebo	4 x 10^8 CFU/day drops of oily suspension	7 days	*L. reuteri* plus rehydration therapy was effective in acute diarrhea reducing the frequency, duration and recurrence rate of the disease.

Shornikova et al [24]	86 children (6-36 months) positive for rotavirus<br>43 probiotics vs 43 placebo	either 10^10 or 10^7 CFU/daily	5 days	It shortens the duration of acute watery diarrhea with a dose-related effect.
Dinleyici et al [25]	64 children (32 probiotics + oral rehydration vs 32 oral rehydration)	1×10^8 CFU/day + oral rehydration solution	5 days	It reduces the duration of acute diarrhea in hospitalized children.
Urbańska et al [26]	review including 1229 infants and children up to 18 years of age	1x 10^8 to 4 × 10^8 CFU/day	5-7 days	The use of this probiotic may be considered in the management of acute gastroenteritis as an adjunct to rehydration.
Szymański et al [27]	99 children (< 5 years old)	2 × 10^8 CFU	5 days	It, in addition to standard rehydration therapy, reduces the duration of hospitalization but not the duration of diarrhea.
Patro-Golab et al [29]	review of 347 participants (172 probiotic group and 175 controls)	daily doses ranged from 1 × 10^8 CFU (5 days) to 2 × 10^8 CFU (5 days) to 4 × 10^8 CFU (7 days)	5-7 days	Useful and safe for the treatment and prevention of diarrhea, reducing both the diarrhea duration and the intensity of symptoms.
